# Incidence, Predictors, and Outcomes of Acute and Sub-acute Stent Thrombosis after Emergency Percutaneous Coronary Revascularization with Drug-Eluting Stents: A Prospective Observational Study

**DOI:** 10.5334/gh.1112

**Published:** 2022-03-30

**Authors:** Rajesh Kumar, Ali Ammar, Tahir Saghir, Jawaid Akbar Sial, Jehangir Ali Shah, Ashok Kumar, Abdul Hakeem Shaikh, Abdul Samad Achakzai, Nadeem Qamar, Musa Karim

**Affiliations:** 1 National Institute of Cardiovascular Diseases (NICVD), Karachi, PK; 2 National Institute of Cardiovascular Diseases (NICVD), Hyderabad, PK

**Keywords:** STEMI, primary PCI, DES, acute, sub-acute, stent thrombosis, MACE

## Abstract

**Background:**

Stent thrombosis (ST) remains the most feared complication of percutaneous coronary intervention (PCI). Therefore, this study aimed to determine acute and sub-acute ST incidence, predictors, and outcomes after primary PCI.

**Methods:**

This prospective observational study included patients who had undergone primary PCI at a tertiary care cardiac center. All the patients were followed at 30-days of index hospitalization for the incidence of acute or sub-acute ST. ST was further categorized as definite, probable, or possible per the Academic Research Consortium definition. All the survivors of ST were followed after 6-months for the incidence of major adverse cardiovascular events.

**Results:**

An aggregate of 1756 patients were included with 79% (1388) male patients and mean age was 55.59 ± 11.23 years. The incidence of ST was 4.9% (86) with 1.3% (22) acute and 3.6% (64) sub-acute. ST was categorized as definite in 3.3% (58) and probable in 1.6% (28). Independent predictor of ST were observed to be male gender (odds ratio (OR); 2.51 [1.21–5.2]), left ventricular end-diastolic pressure ≥20 mmHg (OR; 2.55 [1.31–4.98]), and pre-procedure thrombolysis in myocardial infarction (TIMI) flow 0 (OR; 3.27 [1.61–6.65]). Cumulative all-cause mortality among patients with ST after 164.1 ± 76.2 days was 46.5% (40/86).

**Conclusion:**

We observed a substantial number of patients vulnerable to the acute or sub-acute ST after primary PCI. Male gender, LVEDP, pre-procedure TIMI flow grade can be used to identify and efficiently manage highly vulnerable patients.

## Introduction

Percutaneous coronary intervention (PCI) has dramatically improved in terms of safety and efficiency in the last four decades due to numerous hurdles being addressed. Stent thrombosis (ST) has been considered the significant post-procedural complication of PCI since the introduction of coronary artery stents, with frequently adverse clinical outcomes and high in-hospital and long-term death rates [1]. A lot has changed in terms of timing, occurrence, and preventative tactics of ST from the initial days of bare-metal stents (BMS) to the modern drug-eluting stents (DES) era. In particular, one of the significant advancements in interventional cardiology was the introduction of DES [2]. The use of DES has been shown to reduce in-stent restenosis and subsequent target lesion revascularization with initial increased risk of acute ST [3]. These developments were in keeping with modifications and advancements in PCI adjunctive medication and construction and design of stent, along with an improved comprehension of risk factors and the pathophysiology of ST. Nonetheless, deaths due to ST are still high and range from 11–42% depending on the various patient and system-related factors such as comorbidities, left ventricular function, and level of compliance to the dual antiplatelet treatment (DAPT) [4]. The effective management of ST is an understudied subject in interventional cardiology; therefore prevention and identification of the associated technical and clinical factors remain the pillars of ST care.

The risk of ST observed to be significantly higher in individuals with acute ST-elevation myocardial infarction (STEMI) in comparison to individuals with stable coronary artery diseases (CAD), and various lesion-related, patient-related, procedural, and post-procedural aspects were linked to the increased risk of ST, comprising of lesion features, stent type, and thrombus burden [5,6]. The major risk factors of ST in the initial days of percutaneous stent implantation were technical flaws in the index PCI procedure. Technical shortcomings linked to the increased incidence of ST include residual stenosis, dissection, stent under-sizing, and sub-optimal final coronary flow [7]. Selection of type of antithrombotic medication during a procedure is another major factor of clinical outcomes such as ST. To a certain degree bleeding and ST complications are minimized with the use of DAPT and selection of suitable ADP-receptor inhibitor [3,8].

Regardless of any instrument or related procedural factors, the patient itself is without a doubt the most unpredictable risk factor of ST. Baseline medical history, impending surgeries, antiplatelet drug resistance, bleeding concerns, and other social elements that may impact medication compliance are just a few of the stumbling stones that might lead to an increased risk of ST. Even though several studies have been reported but available data are skewed to specific geographies, data from the South Asian region, especially Pakistan, are almost ceased to exist. To keep up the pace of developments in stent technology, research work should continue to improve our understanding of ST’s underlying mechanism and determinants. Therefore, this study was conducted to identify the occurrence rate of stent thrombosis and determine the factors that may predispose to stent thrombosis even with modern drug-eluting stents (DES) among patients with STEMI undergoing primary PCI at a tertiary care cardiac center of Karachi, Pakistan.

## Material and Methods

### Participants

This study was conducted at the National Institute of Cardiovascular Diseases (NICVD), Karachi, Pakistan, the largest public-sector cardiac hospital in the country, during the study period of August 2020 and July 2021. The ethical review committee of the hospital approved the study (ERC-30/2020). A prospectively selected cohort of consecutive patients with STEMI undergoing primary PCI were recruited for this study. Informed consent for participation was obtained from all the recruited patients. Inclusion criteria for the study were all adult (≥18 years) patients who presented to the emergency department with chest pain within 12 hours, except cardiogenic shock who were enrolled irrespective of time delay, diagnosed with STEMI, and underwent primary PCI at the catheterization laboratory. Patients in whom plain old balloon angioplasty (POBA) done or DES was not placed for any reason, and patients who did not give consent for participation in the study were excluded from this study. This study was conducted during the ongoing COVID-19 pandemic. However, the hospital’s emergency services remained unaltered with standard operating procedures (SOPs) in place for all patient-to-physician interactions.

### Data acquisition and management

The consultant cardiologists performed all the primary PCI procedures as per the current clinical practice guidelines [9]. In-hospital and at discharge pharmacological management was as per the recommendations of clinical practice guidelines. As per the hospital policy, each patient enrolled in the study received initial doses of aspirin, clopidogrel, unfractionated heparin, and bolus dose of glycoprotein inhibitors (IIb/IIIa) (only in patients with short ischemic time and high thrombus burden), the dosage of all the medications were as per the guidelines recommendations. The use of Ticagrelor was limited to patients with stent thrombosis, high-risk anatomy, or diabetes due to cost-effectiveness. DES, mainly resolute onyx DES, was placed during primary PCI procedures as a routine institutional practice.

Data for the study was obtained from all the patients using predefined structured proforma. Pre-procedure clinical and demographic factors included age, gender, duration of symptoms (i.e. total ischemic time), presenting diagnosis (such as type of STEMI, Killip class, arrhythmias, and cardiac arrest on arrival), risk factors (included hypertension, smoking, diabetes mellitus, history of cerebrovascular accident (CVA)/transient ischemic attack (TIA), chronic kidney disease (CKD), and prior myocardial infarction), and baseline vitals and laboratory investigations (included systolic blood pressure (SBP), heart rate (HR), random blood glucose (RBS), hemoglobin (Hb), neutrophil count, platelet count, serum creatinine). Procedure-related factors and angiographic characteristics included access site for the procedure, use of intra-aortic balloon pump (IABP), temporary pacemaker (TPM) implantation, pre-dilatation, use of non-compliant (NC) balloon, left ventricular ejection fraction (LVEF), left ventricular end-diastolic pressure (LVEDP), the number of vessels involved, infarct-related artery, pre-and post-procedure thrombolysis in myocardial infarction (TIMI) flow grade, thrombus grade, vessel diameter, the total length of the lesion, and post-procedure outcome and complications (intra-procedure slow-flow/no-reflow, contrast-induced nephropathy (CIN), arrhythmias, access site complications, bleeding (required transfusion), cardiogenic shock (CS), stroke, re-infarction, in-hospital mortality). All the patients were followed at 30-days of index hospitalization for the incidence of acute or sub-acute ST. All the survivors of ST were followed after 6-months for the incidence of major adverse cardiovascular events (MACE).

### Definitions

STEMI was diagnosed based on symptoms at presentation and a 12-lead electrocardiogram (ECG) as per the fourth universal definition of myocardial infarction [10]. ST was defined and categorized as definite (angiographic confirmation), probable (unexplained death or MI of the stented vessel territory within 30 days), or possible (unexplained death after 30 days with no autopsy confirmation) as per the Academic Research Consortium (ARC) definition [11]. ST was categorized as acute if it occurred within 24 hours of stent implant and sub-acute if it occurred within 30 days of the index procedure. MACE at six months was defined as the occurrence of any of the all-cause mortality, including cardiac- and non-cardiac mortality, CVA/TIA, hospitalization due to heart failure, or myocardial infarction needing revascularization. Cardiac mortality was defined as death due to myocardial ischemia and infarction, heart failure, and sudden cardiac arrest with known or unknown cause.

### Data analysis

IBM SPSS version 21.0 was used to analyze collected data. Patients were categorized into two groups as with and without ST (either acute or sub-acute). Two groups were compared for the demographic distribution, clinical profile, procedural characteristics, and post-procedure outcomes and complications by applying appropriate independent-sample t-test/Mann-Whitney U test or Chi-square test/Likelihood Ratio test/Fisher’s Exact test. Continuous variables that failed to comply with the assumption of normality were expressed as median [interquartile range (IQR)], and Mann-Whitney U test was used for the comparison; otherwise, mean ± standard deviation (SD) was computed, and an independent-sample t-test was applied. At the same time, the Chi-square test was used to compare categorical variables between the two groups. Fisher’s Exact test or Likelihood Ratio test was considered when the expected cell count was less than five to compare the categorical variable with two and more than two levels, respectively. Association between the incidence of ST and various clinical and demographic factors was assessed by conducting univariate and multivariable binary logistic regression analysis. All the factors with a p-value of less than 0.20 in the univariate analysis were taken as predictor variables in the multivariable regression analysis. Odds ratio (OR) along with 95% confidence interval (CI) were obtained, and statistical significance criteria were taken as a p-value of ≤ 0.05.

## Results

An aggregate of 1756 patients were included with 79% (1388) male patients and mean age was 55.59 ± 11.23 years. The incidence of ST was 4.9% (86) with 1.3% (22) acute and 3.6% (64) sub-acute. ST was categorized as definite in 3.3% (58) and probable in 1.6% (28). Incidence of ST was linked to older age (58.45 ± 11.16 vs. 55.44 ± 11.22; p = 0.015), elevated random glucose level (168.5 [260–137] vs. 158 [211–130]; p = 0.023), elevated neutrophil count (14.77 ± 16.94 vs. 11.79 ± 11.3; p = 0.021), high killip class (19.8% [17] vs. 11% [183]), intubation (22.1% [19] vs. 13.4% [223]; p = 0.022), cardiac arrest on arrival (10.5% [9] vs. 4.3% [72]; p = 0.015), use of IABP (11.6% [10] vs. 4.4% [74]; p = 0.007), hypertension (72.1% [62] vs. 55.7% [931]; p = 0.003), diabetes (48.8% [42] vs. 36.2% [605]; p = 0.018), and prior MI (53.5% [46] vs. 5.3% [88]; p < 0.001) (***Table 1***).

**Table 1:** Comparison of demographic factors, presentation, risk factors, baseline vitals, and laboratory investigations between patients with and without stent thrombosis.


CHARACTERISTICS	TOTAL	STENT THROMBOSIS		P-VALUE

		NO	YES	

**Total (N)**	**1756**	**1670 (95.1%)**	**86 (4.9%)**	**–**

**Gender**				

Male	79% (1388)	78.7% (1314)	86% (74)	0.102^a^

Female	21% (368)	21.3% (356)	14% (12)	

**Age (years)**	55.59 ± 11.23	55.44 ± 11.22	58.45 ± 11.16	0.015^b^

<45 years	14.7% (258)	15% (251)	8.1% (7)	0.072^a^

45 to 64 years	61% (1072)	61.1% (1021)	59.3% (51)	

≥65 years	24.3% (426)	23.8% (398)	32.6% (28)	

**Total ischemic time (minutes)**	355 (490–240)	355 (490–240)	330 (570–240)	0.748^c^

**Systolic blood pressure (mmHg)**	131.6 ± 25.9	131.6 ± 25.9	131.8 ± 26.8	0.963^b^

**Heart rate (bpm)**	85.4 ± 20.3	85.3 ± 20.3	89.2 ± 20.6	0.080^b^

**Random glucose level (mg/dL)**	160 (213–130)	158 (211–130)	168.5 (260–137)	0.023^c^

**Hemoglobin level (mg/dL)**	13.64 ± 1.93	13.64 ± 1.93	13.55 ± 1.94	0.682^b^

**Neutrophil count (cells/µL)**	11.94 ± 11.65	11.79 ± 11.3	14.77 ± 16.94	0.021^b^

**Platelet count (cells/µL)**	242.8 (276.5–195)	242.8 (277–194)	242.8 (254–211)	0.665^c^

**Serum creatinine (mg/dL) on arrival**	1.03 ± 0.44	1.02 ± 0.44	1.1 ± 0.43	0.156^b^

**Killip class**				

I	75.5% (1326)	76.3% (1274)	60.5% (52)	0.014^d^

II	13.1% (230)	12.8% (213)	19.8% (17)	

III	7.3% (128)	6.9% (116)	14% (12)	

IV	4.1% (72)	4% (67)	5.8% (5)	

**Type of myocardial infarction (MI)**				

Anterior	55.5% (974)	55.3% (923)	59.3% (51)	0.058^d^

Inferior	16.3% (286)	16.5% (275)	12.8% (11)	

Inferior with RV	18.2% (320)	18.1% (302)	20.9% (18)	

Inferio-posterior	8.4% (147)	8.6% (143)	4.7% (4)	

Lateral	1.7% (29)	1.6% (27)	2.3% (2)	

**Aborted myocardial infarction**	1.9% (34)	2% (34)	0% (0)	0.409^e^

**Intubated**	13.8% (242)	13.4% (223)	22.1% (19)	0.022^a^

**Arrhythmias on arrival**	14.5% (254)	14.4% (240)	16.3% (14)	0.624^a^

**Cardiac arrest on arrival**	4.6% (81)	4.3% (72)	10.5% (9)	0.015^e^

**Co-morbid conditions**				

Hypertension	56.5% (993)	55.7% (931)	72.1% (62)	0.003^a^

Smoking	31.7% (556)	32.2% (538)	20.9% (18)	0.028^a^

Diabetes mellitus	36.8% (647)	36.2% (605)	48.8% (42)	0.018^a^

Prior history of CVA/TIA	2% (35)	1.9% (32)	3.5% (3)	0.244^e^

Chronic kidney disease	1.3% (22)	1.3% (22)	0% (0)	0.622^e^

Prior myocardial infarction	7.6% (134)	5.3% (88)	53.5% (46)	<0.001^a^


CVA = cerebrovascular accident, TIA = transient ischemic attack. *^a^* Chi-square test. *^b^* Independent-sample t-test. *^c^* Mann-Whitney U test. *^d^* Likelihood Ratio test. *^e^* Fisher’s Exact test.

More common angiographic characteristics among patients with ST were elevated LVEDP (22.3 ± 7.8 vs. 19.5 ± 6.5; p < 0.001), pre-TIMI grade 0 (81.4% [70] vs. 61.8% [1032]), thrombus grade III or IV (89.5% [77] vs. 79.3% [1325]), post-procedure sub-optimal flow (22.1% [19] vs. 9.8% [164]), and intra-procedure slow-flow/no-reflow (41.9% [36] vs. 29.8% [497]; p = 0.017) (***Table 2***).

**Table 2:** Comparison of angiographic and procedural characteristics, post-procedure complications, and in-hospital outcomes between patients with and without stent thrombosis.


CHARACTERISTICS	TOTAL	STENT THROMBOSIS		P-VALUE

		NO	YES	

**Total (N)**	**1756**	**1670 (95.1%)**	**86 (4.9%)**	**–**

**Access for procedure**				

Radial	68.2% (1197)	68.8% (1149)	55.8% (48)	0.008^d^

Femoral	30.4% (534)	29.7% (496)	44.2% (38)	

Switchover	1.4% (25)	1.5% (25)	0% (0)	

**LV end-diastolic pressure (mmHg)**	19.7 ± 6.6	19.5 ± 6.5	22.3 ± 7.8	<0.001^b^

<20 mmHg	48.1% (845)	49% (819)	30.2% (26)	0.001^a^

≥20 mmHg	51.9% (911)	51% (851)	69.8% (60)	

**LV ejection fraction (%)**	39.5 ± 9.3	39.6 ± 9.3	38.3 ± 8.7	0.186^b^

**Temporary pacemaker implanted**	6.7% (118)	6.8% (113)	5.8% (5)	0.731^a^

**Intra-aortic balloon pump used**	4.8% (84)	4.4% (74)	11.6% (10)	0.007^d^

**Number of vessels involved**				

Single vessel disease	36.7% (645)	37.2% (621)	27.9% (24)	0.101^a^

Two vessel disease	34.2% (600)	34.2% (571)	33.7% (29)	

Three vessel disease	29.1% (511)	28.6% (478)	38.4% (33)	

**Recanalised vessel**	5% (87)	5.1% (85)	2.3% (2)	0.438^e^

**Culprit coronary artery**				

Left main	1.3% (23)	1.3% (22)	1.2% (1)	0.224^d^

LAD; Proximal	36% (633)	35.6% (595)	44.2% (38)	

LAD; Non-Proximal	19% (334)	19.3% (322)	14% (12)	

Right coronary artery	31.3% (550)	31.3% (522)	32.6% (28)	

Left circumflex	11.3% (198)	11.6% (193)	5.8% (5)	

Diagonal	1% (18)	1% (16)	2.3% (2)	

**Pre-procedure TIMI flow**				

0	62.8% (1102)	61.8% (1032)	81.4% (70)	0.003^a^

I	9.6% (168)	9.8% (164)	4.7% (4)	

II	15.6% (274)	16.1% (269)	5.8% (5)	

III	12.1% (212)	12.3% (205)	8.1% (7)	

**Thrombus Grade**				

G1	3.1% (55)	3.1% (52)	3.5% (3)	0.002^d^

G2	3.5% (62)	3.7% (61)	1.2% (1)	

G3	13.5% (237)	13.9% (232)	5.8% (5)	

G4	17.1% (300)	17.5% (293)	8.1% (7)	

G5	62.8% (1102)	61.8% (1032)	81.4% (70)	

**Pre-balloon used**				

Not done	53% (931)	53.4% (891)	46.5% (40)	0.179^d^

Dottering	3% (53)	3.1% (52)	1.2% (1)	

Dilatation	44% (772)	43.5% (727)	52.3% (45)	

**Non-compliant (NC) balloon used**	81.4% (1429)	81.6% (1363)	76.7% (66)	0.258^a^

**Mean vessel diameter**	3.5 ± 0.3	3.5 ± 0.3	3.5 ± 0.3	0.645^b^

**Total lesion length**	27.9 ± 11.9	27.8 ± 11.9	28.3 ± 12.3	0.702^b^

**Fluoroscopic-time (minutes)**	15.1 ± 8.3	15.1 ± 8.3	14.6 ± 7.4	0.529^b^

**Contrast volume (ml)**	120.3 ± 37.4	120.6 ± 37.3	114.5 ± 38.4	0.137^b^

**Intra-procedure slow-flow/no-reflow**	30.4% (533)	29.8% (497)	41.9% (36)	0.017^a^

**Post-procedure TIMI flow**				

0	0.3% (6)	0.4% (6)	0% (0)	0.006^d^

I	1.5% (27)	1.4% (23)	4.7% (4)	

II	8.5% (150)	8.1% (135)	17.4% (15)	

III	89.6% (1573)	90.2% (1506)	77.9% (67)	

**Reperfusion arrhythmias**	23.9% (419)	24.1% (403)	18.6% (16)	0.241^a^

**Post-procedure complications and in-hospital outcomes**				

In-hospital death	3.5% (61)	3.1% (51)	11.6% (10)	0.001^e^

Contrast induced nephropathy	9.6% (169)	9.6% (160)	10.5% (9)	0.786^a^

Arrhythmias	3.6% (63)	3.3% (55)	9.3% (8)	0.101^e^

Access site complications	0.7% (13)	0.8% (13)	0% (0)	>0.999^e^

Bleeding	0.9% (16)	0.8% (13)	3.5% (3)	0.040^e^

Cardiogenic shock	3.9% (68)	3.6% (60)	9.3% (8)	0.016^e^

CVA/TIA	0.2% (4)	0.2% (4)	0% (0)	>0.999^e^

Re-infarction	1% (18)	0.4% (6)	14% (12)	<0.001^e^


LV = left ventricular, LAD = left anterior descending artery, TIMI = thrombolysis in myocardial infarction, CVA = cerebrovascular accident, TIA = transient ischemic attack. *^a^* Chi-square test. *^b^* Independent-sample t-test. *^c^* Mann-Whitney U test. *^d^* Likelihood Ratio test. *^e^* Fisher’s Exact test.

On multivariable analysis, independent predictor of ST was observed to be male gender with adjusted OR of 2.51 [95% CI: 1.21–5.2] LVEDP ≥ 20 mmHg with adjusted OR of 2.55 [95% CI: 1.31–4.98], and pre-procedure TIMI flow grade 0 with adjusted OR of 3.27 [95% CI: 1.61–6.65] (***Table 3***).

**Table 3:** Univariate and multivariable logistic regression analysis for stent thrombosis.


FACTORS	UNIVARIATE		MULTIVARIABLE	
	
	OR [95% CI]	P-VALUE	OR [95% CI]	P-VALUE

Male	1.67 [0.90–3.11]	0.105	2.51 [1.21–5.2]	0.013*

Age (years)	1.02 [1.00–1.04]	0.016*	1.01 [0.99–1.04]	0.248

Total ischemic time (minutes)	1.00 [1.00–1.00]	0.698	–	–

Systolic blood pressure (mmHg)	1.00 [0.99–1.01]	0.963	–	–

Heart rate (bpm)	1.01 [1.00–1.02]	0.080	1.01 [1.00–1.02]	0.148

Random glucose level (mg/dL)	1.00 [1.00–1.00]	0.056	1.00 [1.00–1.00]	0.410

Hemoglobin level (mg/dL)	0.98 [0.87–1.09]	0.682	–	–

Neutrophil count (cells/µL)	1.01 [1.00–1.03]	0.026*	1.01 [1.00–1.03]	0.147

Platelet count (cells/µL)	1.00 [1.00–1.00]	0.378	–	–

Serum creatinine (mg/dL) on arrival	1.32 [0.89–1.94]	0.162	1.09 [0.65–1.83]	0.751

Killip class III/IV	2.00 [1.15–3.48]	0.014*	1.00 [0.37–2.72]	0.999

Intubated	1.84 [1.08–3.12]	0.024*	0.70 [0.26–1.89]	0.487

Arrhythmias on presentation	1.16 [0.64–2.09]	0.624	–	–

Cardiac arrest	2.59 [1.25–5.38]	0.01*	1.55 [0.54–4.4]	0.415

Hypertension	2.05 [1.27–3.32]	0.003*	1.65 [0.94–2.91]	0.084

Smoking	0.56 [0.33–0.95]	0.03*	0.71 [0.38–1.31]	0.276

Diabetes mellitus	1.68 [1.09–2.59]	0.019*	1.05 [0.56–1.95]	0.888

LV end-diastolic pressure ≥ 20 mmHg	2.22 [1.39–3.55]	<0.001*	2.55 [1.31–4.98]	0.006*

LV ejection fraction (%)	0.98 [0.96–1.01]	0.186	1.03 [0.99–1.06]	0.140

Intra-aortic balloon pump used	2.84 [1.41–5.71]	0.003*	1.32 [0.46–3.72]	0.606

Triple vessel disease	1.55 [0.99–2.43]	0.054	1.18 [0.68–2.04]	0.559

Culprit left main or proximal LAD	1.42 [0.92–2.19]	0.118	1.07 [0.58–1.95]	0.835

Pre TIMI flow grade 0	2.70 [1.56–4.70]	<0.001*	3.27 [1.61–6.65]	0.001*

Thrombus grade ≥ 4	1.49 [0.54–4.13]	0.446	–	–

Pre-dilatation	1.42 [0.92–2.20]	0.111	1.26 [0.75–2.1]	0.383

Non-compliant balloon used	0.74 [0.44–1.24]	0.259	–	–

Mean vessel diameter (mm)	1.16 [0.62–2.17]	0.645	–	–

Total lesion length (mm)	1.00 [0.99–1.02]	0.701	–	–

Intra-procedure slow-flow/no-reflow	1.70 [1.09–2.64]	0.018*	0.66 [0.32–1.36]	0.264

Final sub-optimal TIMI flow (<3)	2.60 [1.53–4.44]	<0.001*	2.32 [0.99–5.44]	0.053

Contrast induced nephropathy	1.10 [0.54–2.24]	0.786	–	–

Arrhythmias	3.01 [1.39–6.54]	0.005*	1.39 [0.49–3.95]	0.533

Bleeding	4.61 [1.29–16.48]	0.019*	3.38 [0.71–16.03]	0.124

Cardiogenic shock	2.75 [1.27–5.96]	0.01*	1.37 [0.42–4.45]	0.601


OR = odds ratio, CI = confidence interval, LV = left ventricular, LAD = left anterior descending artery, TIMI = thrombolysis in myocardial infarction. * Significant at 5%.

After a mean follow-up of 164.1 ± 76.2 days, cumulative all-cause mortality among patients with ST was 46.5% (40/86), cardiac mortality in 43% (37/86), MI needing revascularization in 84.9% (73/86), hospitalization due to HF in 15.1% (13/86), and stroke/CVA in one (1.2%) patient.

## Discussion

Even though the cardiology community is more aware of the complications of ST, the lack of knowledge presently accessible on risk stratification and the usefulness of particular strategies for the management is astonishing [1]. Less frequent incidence of the complication itself is one of the major reasons that substantial studies of ST have been difficult to conduct. The studies that have been done to this point have applied predominantly, if not completely, the older generation stents. Hence in this study, we aimed at determining the incidence of ST and its determinants along with associated short-term outcomes. The incidence of ST was 4.9% among patients with STEMI undergoing primary PCI, with 1.3% acute and 3.6% sub-acute in nature. According to the ARC definition, 3.3% of the ST events were definite, and the remaining 1.6% were probable ST. In this study male gender (2.51 [1.21–5.2]), LVEDP ≥ 20 mmHg (2.55 [1.31–4.98]), and pre-procedure TIMI flow grade 0 (3.27 [1.61–6.65]) were observed to be significant independent predictors of acute or sub-acute ST after primary PCI. Cumulative all-cause mortality among patients with ST after 164.1 ± 76.2 days was 46.5%, re-MI needing revascularization was 84.9%, hospitalization due to HF was 15.1%, and stroke/CVA was observed in one (1.2%) patient.

The reported incidence rate of ST varies significantly from 5.9% to as high as 18.8%, depending on the type and generation of stent implanted [4]. The incidence rate in our population was relatively lower than that reported in previous studies from various other parts of the world [4] and from our population. An incidence rate of 5.8% was reported previously [12]. Similar to our study, Lim S et al., [13] reported definite or probable ST in 3.7% out of 4,748 patients who went through PCI after AMI during a median follow-up of 4 and half years, including 1.0% with early ST, 0.9% with late ST, and 2.0% with very late ST. During a median follow-up of 30.9 months, a cumulative incidence rate of angiographically confirmed ST was 2.1% in a study from the Dutch Stent Thrombosis Registry [7]. A study based on data from a multicenter Spanish registry reported an ST incidence rate of 2% in 3 years after the deployment of DES [14]. A study from North Carolina reported an ST rate of 2.7% at 30 days of primary PCI [15].

ST’s pathophysiology is not very well understood; comorbidities, e.g. diabetes, stent design, lesion morphology, patients’ adherence to the antiplatelet therapy, and high on-treatment platelet reactivity are all can be possible causes of ST [7]. There have been various patient-related, device-related, lesion-related, and practice-related determinants of ST discovered in literature so far. Patient and lesion-related factors are two of the most critical determinants of ST [16].

Notably, duration between deployment of stent and occurace of ST appears to be a major confounding factor in determining the role of various clinical factors for the prediction of ST. For example, diabetes and kidney failure appear to be strong predictors of late ST, on the other hand, small vessel disease and in-stent restenosis appear to be strongly associated with the incidence of early ST [17]. Align with these observations, three factors, i.e., LVEDP ≥ 20 mmHg, pre-procedure TIMI flow grade, and gender of the patient, were found to be independent predictors of acute or sub-acute ST after primary PCI.

Among the procedure-related predictors of ST, a study on 6,545 PCI patients by Rozemeijer R et al. [18] reported that the factors associated with an increased risk of ST include angiographic stent under expansion, exposed edge segmentation, lesion complexity, and residual lesion in the range of 5 mm of the stent. According to optical coherence tomography and intravascular ultrasonography studies [19,20,21], short stent expansion spans 20–40%, and stent edge dissection spans 10–50%. The necessity of proper stent deployment and size, specifically in individuals with complicated lesions, has been addressed in several publications [22].

Among patients’ related predictors, diabetes, kidney failure, and STEMI were independent sub-acute ST indicators in a multicenter registry from Spain [14]. Diabetics, in particular, were observed to have twice the risk of a rate of ST compared to non-diabetics [23]. No-reflow phenomenon, reduced left ventricular ejection fraction, anemia, and an average stent diameter of less than 3.0 mm were all independent indicators of ST [13]. In line with these findings, on univariate analysis in this study, we observed various patients, lesion, and procedure-related factors associated (p-value ≤ 0.005) with increased risk of acute or sub-acute ST, which included increasing age of the patient, elevated neutrophil count, Killip class III/IV at presentation, need of intubation, cardiac arrest at presentation, hypertensive, diabetic, use of intra-aortic balloon pump, intra-procedure slow-flow/no-reflow, development of post-procedure arrhythmias, and cardiogenic shock. Prior research is inconsistent regarding predictors of ST, the range of predictors reported in various studies include renal insufficiency, insulin-dependent diabetes mellitus, history of congestive heart failure, history of PCI, prior infarction, Killip classes III to IV, STEMI, ST-segment deviation ≥1 mm, reperfusion time ≤2 hours, baseline platelet count, and baseline hemoglobin [13,15]. Secondly, the number of stents implanted, the extent of CAD, angiographic aneurysm, bifurcation lesion, angiographic ulceration, stent diameter and size, low baseline TIMI flow, high thrombus grade, and pre-and peri-procedure clopidogrel use are a few of the several procedures- or treatment-related factors associated with increased risk of ST [12,13,15,24]. However, in our study, a number of significant clinical predictors failed to attain the required statistical significance in multivariable analysis, this could be partly attributed to the distinctiveness of our population and lack of exhaustiveness of data at disposal for the analysis due to observational nature of study.

Guidelines on optimal management of ST are not well established; therefore, early detection and preventive measures remain the cornerstones of ST management. Various preventive tactics have been adopted but no groundbreaking success so far. For instance, Clemmensen P et al. [5] analyzed from EURO MAX (European Ambulance Acute Coronary Syndrome Angiography) trial and endorses both new oral P2Y12 inhibitors and low dose (0.25 mg/kg/h) bivalirudin infusion proved to be effective in minimizing the risk of ST. The study further reported that the risk of development of acute ST following primary PCI is restricted to the first few hours. Although continuing the bivalirudin infusion at the complete dose for the initial several hours following primary PCI was not linked to an increased risk of ST whereas retaining lower bleeding events, recommending that this method could significantly improve primary PCI results.

This study reported data of the largest cohort of STEMI patients ever studied for acute and sub-acute ST incidence in our population. However, certain limitations may limit the generalizability of the study findings, first due to the observational nature of the study design, patient selection bias can be expected. We could not assess the relationship between ST and stent malposition due to a lack of intravascular ultrasound (IVUS) studies. Being a public sector hospital with an institutional policy of free-of-cost primary PCI services, intravascular imaging, such as IVUS/optical coherence tomography, is not a routine practice due to lack of resources. Additionally, data regarding some of the potentially confounding variables, such as number of stents per patient, anatomical complexities, or stenting techniques, were not available for the analysis. Similarly, we were also unable to investigate other probable causes of ST, such as polymer hypersensitivity, delayed arterial wall remedial, strut fracture, and new plaque rupture. Non-adherence to the DAPT can have a significant impact on the incidence of sub-acute ST. However, these factors also could not be formally evaluated, and no conclusion can be made regarding the association between risk of ST and non-adherence to DAPT. Finally, post 30-day follow-up was only conducted for the patients who survived with acute or sub-acute ST. Hence, we could not analyze the impact of ST on short-term MACE and incidence of late and very late ST.

## Conclusion

A substantial number of STEMI patients were vulnerable to the acute or sub-acute ST after primary PCI in the DES era. Male gender, LVEDP ≥ 20 mmHg, and pre-procedure TIMI flow grade were independent predictors of acute or sub-acute ST. Proper identification and efficient management of highly vulnerable patients can reduce the burden of ST and subsequent adverse outcomes.

## Data Accessibility Statement

Data of the study will be available upon request.
